# Low-Dose Alcohol Consumption Protects against Transient Focal Cerebral Ischemia in Mice: Possible Role of PPARγ

**DOI:** 10.1371/journal.pone.0041716

**Published:** 2012-07-27

**Authors:** Hong Sun, Wanfen Xiong, Denise M. Arrick, William G. Mayhan

**Affiliations:** 1 Department of Cellular Biology and Anatomy, Louisiana State University Health Sciences Center-Shreveport, Shreveport, Louisiana, United States of America; 2 Department of Surgery, University of Nebraska Medical Center, Omaha, Nebraska, United States of America; Univ. Kentucky, United States of America

## Abstract

**Background:**

We examined the influence of low-dose alcohol consumption on cerebral ischemia/reperfusion (I/R) injury in mice and a potential mechanism underlying the neuroprotective effect of low-dose alcohol consumption.

**Methodology/Principal Findings:**

C57BL/6 J mice were fed a liquid diet without or with 1% alcohol for 8 weeks, orally treated with rosiglitazone (20 mg/kg/day), a peroxisome proliferator-activated receptor gamma (PPARγ)-selective agonist, or GW9662 (3 mg/kg/day), a selective PPARγantagonist, for 2 weeks. The mice were subjected to unilateral middle cerebral artery occlusion (MCAO) for 90 minutes. Brain injury, DNA fragmentation and nuclear PPARγ protein/activity were evaluated at 24 hours of reperfusion. We found that the brain injury and DNA fragmentation were reduced in 1% alcohol-fed mice compared to nonalcohol-fed mice. Rosiglitazone suppressed the brain injury in nonalcohol-fed mice, but didn't alter the brain injury in alcohol-fed mice. In contrast, GW9662 worsened the brain injury in alcohol-fed mice, but didn't alter the brain injury in nonalcohol-fed mice. Nuclear PPARγ protein/activity at peri-infarct and the contralateral corresponding areas of the parietal cortex was greater in alcohol-fed mice compared to nonalcohol-fed mice. Using differentiated catecholaminergic (CATH.a) neurons, we measured dose-related influences of chronic alcohol exposure on nuclear PPARγ protein/activity and the influence of low-dose alcohol exposure on 2-hour oxygen-glucose deprivation (OGD)/24-hour reoxygenation-induced apoptosis. We found that low-dose alcohol exposure increased nuclear PPARγ protein/activity and protected against the OGD/reoxygenation-induced apoptosis. The beneficial effect of low-dose alcohol exposure on OGD/reoxygenation-induced apoptosis was abolished by GW9662.

**Conclusions/Significance:**

Our findings suggest that chronic consumption of low-dose alcohol protects the brain against I/R injury. The neuroprotective effect of low-dose alcohol consumption may be related to an upregulated PPARγ.

## Introduction

Ischemic stroke is one of the leading causes of death and permanent disability and has limited therapeutic options. Alcohol is one of the most commonly used chemical substances. Increasing evidence suggests that light-moderate alcohol exposure can typically initiate cytoprotective mechanisms [1]. The brain is a major target organ of the actions of alcohol. Epidemiological studies suggest that light-moderate alcohol consumption reduces mortality and infarct volume from ischemic stroke [2], [3]. Recently, a prospective cohort study in men found a beneficial effect of light alcohol consumption on functional outcome from ischemic stroke [4]. However, these epidemiological studies didn't provide detailed information according to ischemic stroke subtype, ischemic duration and ischemic region. In addition, mechanisms underlying neuroprotective effect of light-moderate alcohol consumption are not clear.

Ischemic stroke accounts for approximately 85% of all strokes [5]. Due to the advances in intravascular techniques and thrombolytic agents, transient focal cerebral ischemia has become one of the most common types of ischemic stroke. Unfortunately, there is a paucity of experimental data regarding the influence of alcohol consumption on the consequence of transient focal ischemic stroke. Recently, we found that 8-week low-dose (1% (v/v)) alcohol consumption significantly reduced 2-hour MCAO/24-hour reperfusion-induced brain damage in rats [6]. Thus, our first goal of the present study was to corroborate the neuroprotective effect of low-dose alcohol consumption in a mouse model of transient focal cerebral ischemia.

PPARs are members of the nuclear hormone receptor superfamily of ligand-activated transcription factors. In the central nervous system (CNS), PPARs have been implicated in neural cell differentiation and death as well as in inflammation and neurodegeneration [7]. Pharmacological activation of all PPAR isoforms, but especially of PPARγ, has been demonstrated to protect against focal cerebral I/R injury [8]. Chronic high-dose alcohol consumption has been shown to alter PPARγ expression/activity in organs, tissues and cells [9], [10], [11], [12]. As far as we are aware no studies have reported the influence of chronic alcohol consumption on PPARγ expression/activity in the CNS. Thus, our second goal of the present study was to measure the influence of low-dose alcohol consumption on nuclear PPARγ protein/activity in the cerebral cortex and determine whether the neuroprotective effect of low-dose alcohol consumption is related to an altered nuclear PPARγ protein/activity.

## Results

### Control conditions

There was no significant difference in body weight (nonalcohol: 31.2±0.5 g; nonalcohol+rosiglitazone: 31.6±0.3 g; nonalcohol+GW9662: 31.0±0.2 g; 1% alcohol: 31.6±0.4 g; 1% alcohol+rosiglitazone: 31.5±0.6 g; 1% alcohol+GW9662: 31.4±0.5 g) following feeding 1% (v/v) alcohol diet for 8 weeks and treating with rosiglitazone or GW9662 for 2 weeks. The plasma alcohol concentration in 1% alcohol group at 0.5, 1, 2, and 4 hours after giving alcohol diet was 0.8, 1.0, 0.5 and 0 mM, respectively.

### MCAO/reperfusion-induced brain injury

**Figure 1:**
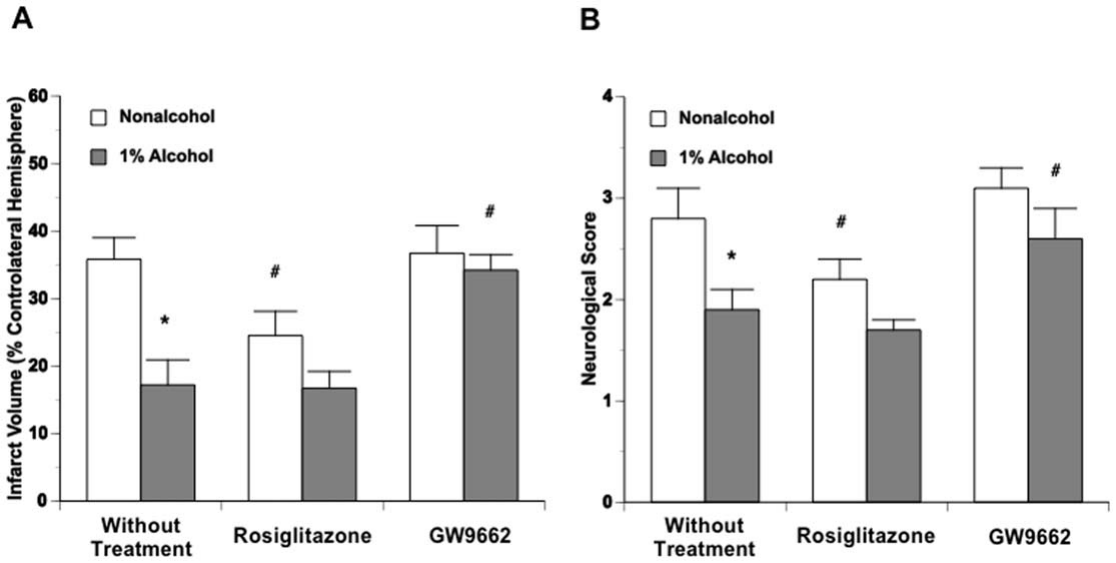
Total infarct volume (A) and neurological score (B) at 24 hours of reperfusion after a 90-minute MCAO in nonalcohol-fed and 1% alcohol-fed mice treated without and with rosiglitazone or GW9662. Values are means ± SE for 6 mice in each group. *P<0.05 vs. Nonalcohol. #P<0.05 vs. Without Treatment.

After the mice were fed with nonalcohol or 1% alcohol diets for 8 weeks and treated with rosiglitazone or GW9662 for 2 weeks, they were subjected to unilateral MCAO for 90 minutes. At 24 hours of reperfusion, mice were neurologically evaluated and sacrificed for measuring infarct volume by TTC staining. The total infarct volume was 35.8±3.2% of contralateral hemisphere in nonalcohol-fed mice. There was a significant reduction in 1% alcohol-fed mice (18.2±3.7%) compared to nonalcohol-fed mice. Rosiglitazone significantly reduced total infarct volume in nonalcohol-fed mice, but did not alter the total infarct volume in 1% alcohol-fed mice. In contrast, GW9662 did not alter the total infarct volume in nonalcohol-fed mice, but significantly increased total infarct volume in 1% alcohol-fed mice (Figure 1A). Consistent with the findings regarding the total infarct volume, the neurological deficits were significantly improved in 1% alcohol-fed mice. In addition, rosiglitazone significantly reduced the neurological deficits in nonalcohol-fed mice, whereas GW9662 significantly worsened the neurological deficits in 1% alcohol-fed mice (Figure 1B).

### Nuclear PPARγ protein and DNA-binding activity of PPARγ in parietal cortex

**Figure 2:**
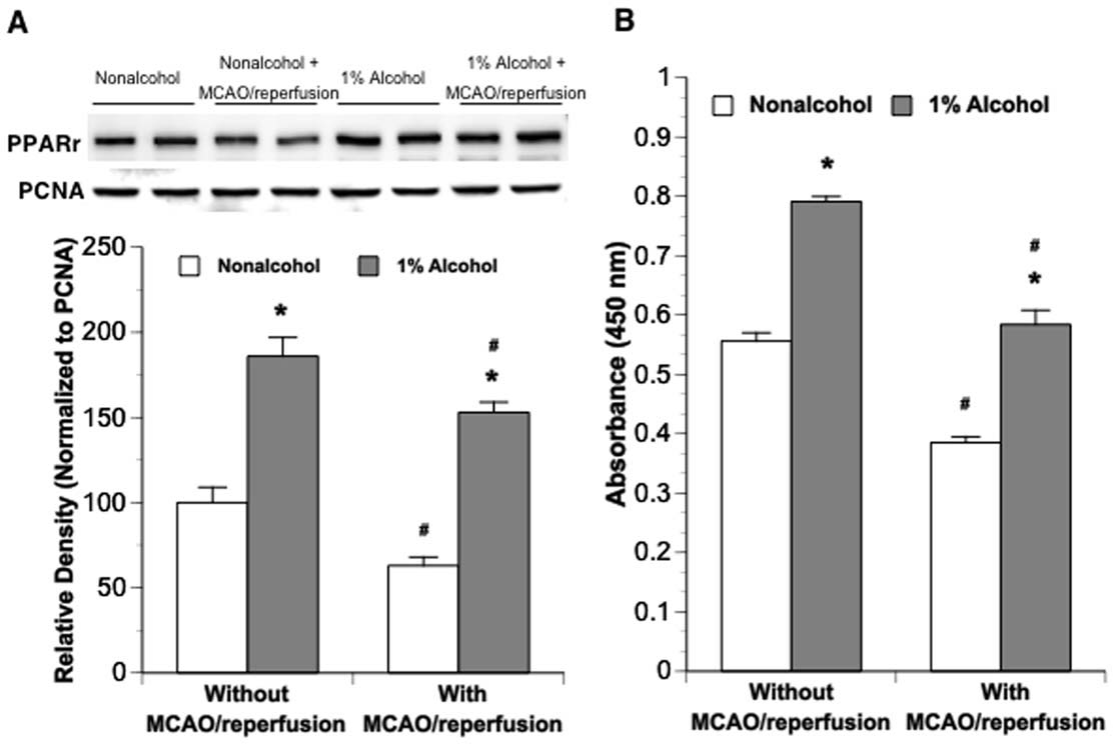
Nuclear protein expression (A) and DNA-binding activity (B) of PPARγ in parietal cortex punched at the peri-infarct and the contralateral corresponding areas of nonalcohol-fed and 1% alcohol-fed mice following a 90-minute MCAO/24-hour reperfusion. Values are means ± SE for 6 mice in each group. *P<0.001 vs. Nonalcohol. #P<0.001 vs. Without MCAO/reperfusion.

**Figure 3:**
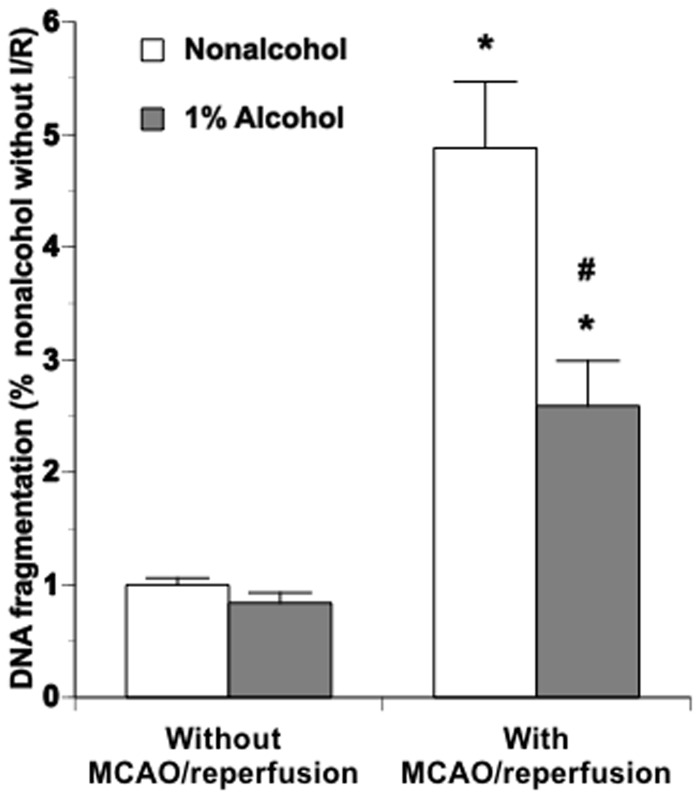
DNA fragmentation in parietal cortex punched at the peri-infarct and the contralateral corresponding areas of nonalcohol-fed and 1% alcohol-fed mice following a 90-minute MCAO/24-hour reperfusion. Values are means ± SE for 6 mice in each group. *P<0.001 vs. Nonalcohol. #P<0.001 vs. Without MCAO/reperfusion.

In a separate group, 8-week nonalcohol-fed and 1% alcohol-fed mice were subjected to unilateral MCAO for 90 minutes. At 24 hours of reperfusion, mice were neurologically evaluated and sacrificed for measuring nuclear PPARγ protein expression (Figure 2A), nuclear PPARγ DNA-binding activity (Figure 2B) and DNA fragmentation (Figure 3). Tissues punched at peri-infarct and the contralateral corresponding areas of the parietal cortex were used. Nuclear PPARγ was significantly upregulated in the contralateral corresponding parietal cortex of 1% alcohol-fed mice. Ninety-minute MCAO/24-hour reperfusion downregulated nuclear PPARγ in both nonalcohol-fed and alcohol-fed mice. However, nuclear PPARγ protein/activity following the MCAO/reperfusion was still significantly greater in alcohol-fed mice compared to nonalcohol-fed mice (Figure 2).

### MCAO/reperfusion-induced DNA fragmentation

There was no significant difference in DNA fragmentation of the parietal cortex between nonalcohol-fed and alcohol-fed groups at basal conditions. MCAO/reperfusion significantly increased DNA fragmentation in both nonalcohol-fed and alcohol-fed mice. However, the magnitude of increase was significantly less in alcohol-fed mice compared to nonalcohol-fed mice (Figure 3).

### Nuclear protein and DNA-binding activity of PPARγ in cultured neurons

**Figure 4:**
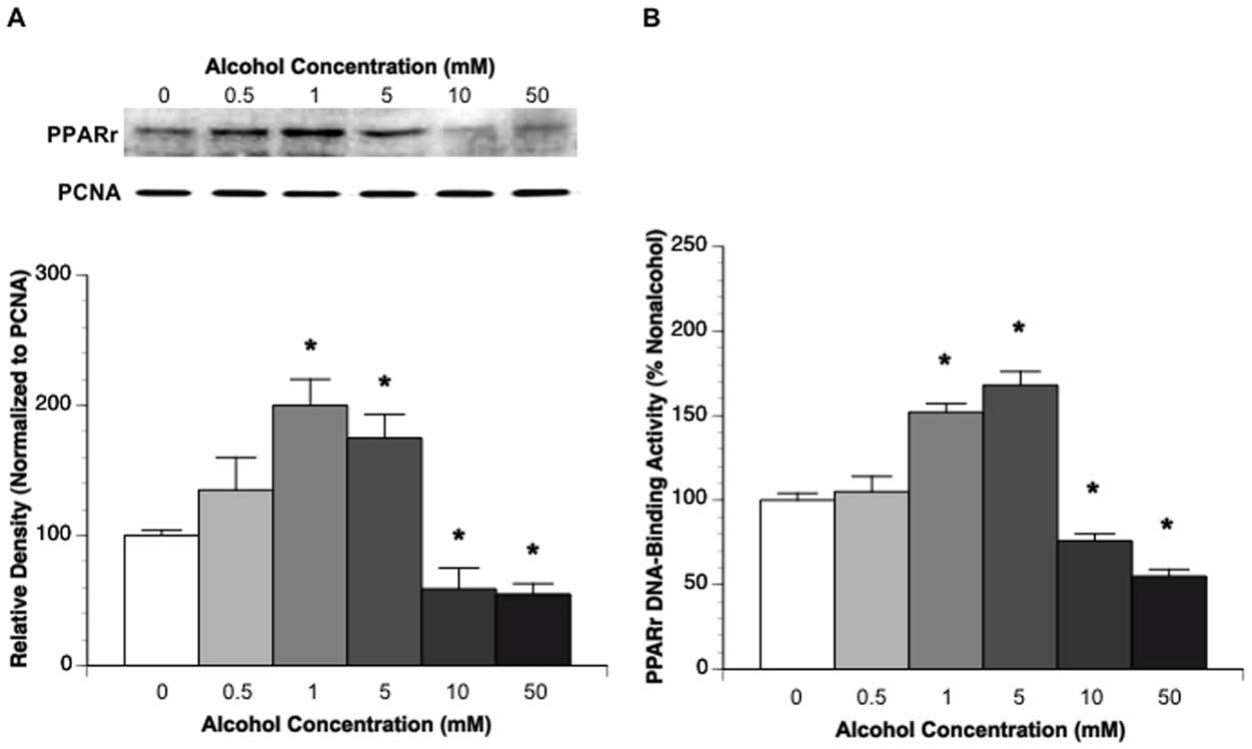
Dose-related influences of chronic alcohol exposure on nuclear protein expression (A) and DNA-binding activity (B) of PPARγ in CATH.a neurons. Values are means ± SE for 6 wells in each group. *P<0.05 vs. 0 mM alcohol.

Dose-related influences of chronic alcohol exposure on nuclear PPARγ protein (Figure 4A) and DNA-binding activity (Figure 4B) in CATH.a neurons were measured. Nuclear PPARγ was significantly upregulated in 7-day 1 mM and 5 mM alcohol-exposed, but downregulated in 7-day 10 mM and 50 mM alcohol-exposed CATH.a neurons.

### OGD/reoxygenation-induced apoptosis

**Figure 5:**
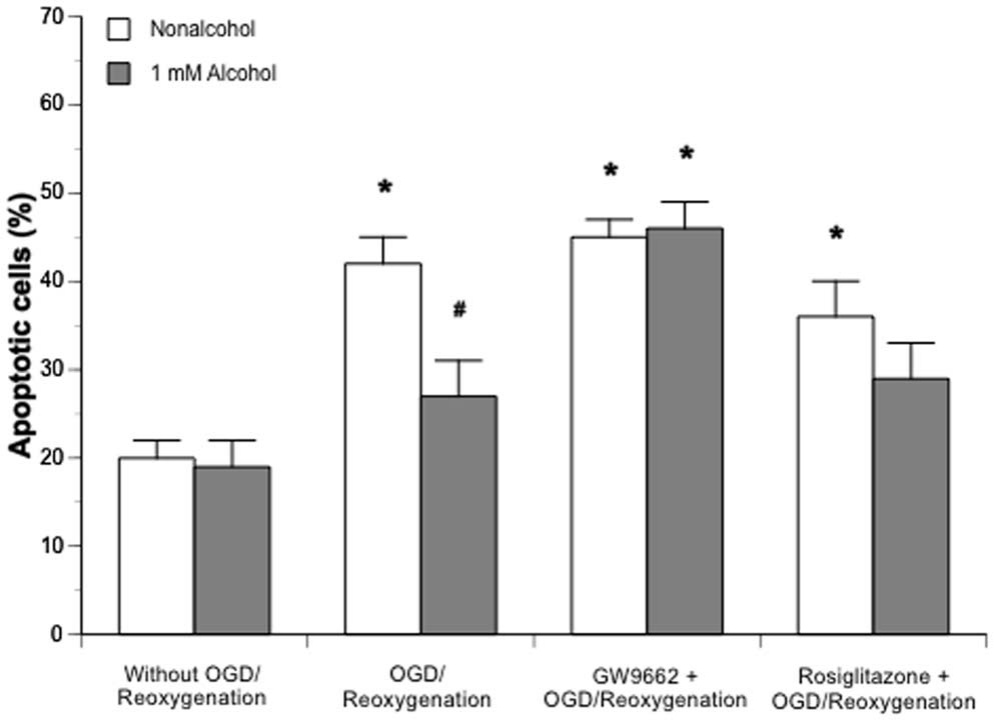
Effect of chronic exposure of 1 mM alcohol on apoptosis in CATH.a neurons following a 2-hour OGD/24-hour reoxygenation. Values are means ± SE for 6 wells in each group. *P<0.01 vs. Without OGD/reperfusion. *P<0.05 vs. Nonalcohol.

Apoptosis following 2-hour OGD/24-hour reoxygenation was measured in CATH.a neurons exposed with 1 mM alcohol alone or in combination with 15 µM GW9662 and 5 µM rosiglitazone for 7 days. Alcohol exposure didn't alter the percentage of apoptotic cells at basal conditions, but significantly reduced the percentage of apoptotic cells following the OGD/reoxygenation. The protective effect of alcohol on OGD/reoxygenation-induced apoptosis was not altered by rosiglitazone, but abolished by GW9662 (Figure 5).

## Discussion

There are several new findings from this study. First, low-dose alcohol consumption significantly reduced cerebral I/R-induced infarct volume, DNA fragmentation and neurological deficits in mice. Second, PPARγ was upregulated in the cerebral cortex of low-dose alcohol-fed mice and low-dose alcohol-exposed CATH.a neurons. Third, treatment with PPARγ-selective antagonist abolished the protective effect of low-dose alcohol on transient focal cerebral ischemia-induced brain injury and OGD/reoxygenation-induced apoptosis. We suggest that the neuroprotective effect of low-dose alcohol consumption may be related to an upregulated PPARγ.

PPARγ is a ligand-activated transcription factor that was originally discovered as a regulator of fatty acid storage and glucose metabolism. Three isoforms, PPARγ1, PPARγ2 and PPARγ3, have been identified. PPARγ1 is expressed nearly in all tissues. PPARγ2 is mostly expressed in adipose tissue. PPARγ3 is mainly expressed in macrophages, large intestine and white adipose tissue. PPARγ form heterodimers with retinoid X receptors (RXRs) in the nucleus and these heterodimers regulate transcription of various genes. PPARγ has been implicated in the pathology of numerous diseases including obesity, diabetes, atherosclerosis and cancer [13]. Recently, PPARγ agonists have been shown to protect the brain against its I/R injury [14], [15]. In the present study, we found that low-dose alcohol consumption increase nuclear PPARγ content/DNA-binding activity in cerebral cortex. In addition, PPARγ antagonist, GW9662, abolished the neuroprotective effect of low-dose alcohol consumption. GW9662 is a potent irreversible antagonist of PPARγ [16]. Although GW9662 binds PPARα and PPARδ it did not alter transcription of full-length PPARα and PPARδ [17], [18]. In addition, the inhibitory effect of GW9662 was much more potent on activated PPARγ than on activated PPARα or PPARδ [19]. To exclude other possible effects of GW9662, we further determined the effect of PPARγ-selective agonist, rosiglitazone. We found that rosiglitazone protected against cerebral I/R injury in nonalcohol-fed mice but not in low-dose alcohol-fed mice. Thus, low-dose alcohol consumption may protect against cerebral I/R injury via an upregulated PPARγ.

Several studies have investigated PPARγ expression and DNA-binding activity in the peri-infarct cortex. Zhao et al. found that PPARγ immunoreactive cells dramatically increased at 12 hours of reperfusion but restored to the normal level at 24 hours of reperfusion in 90-minute MCAO rats [20]. Ou et al. reported that PPARγ mRNA significantly increased at 6 and 24 hours of reperfusion in 180-minute MCAO rats [21]. Victor et al. found that PPARγ mRNA and immunoreactive cells dramatically increased up to 14 days of reperfusion in 120-minute MCAO rats. In contrast, PPARγ DNA-binding activity significantly reduced in peri-infarct cortex [15]. In the present study, we found that both nuclear PPARγ protein and DNA-binding activity were significantly reduced at 24 hours of reperfusion, indicating that suppressed PPARγ DNA-binding activity may be related to a reduced PPARγ protein in the nucleus. In addition to the nucleus, PPARγ also expresses in the cytoplasm [22]. Thus, upregulated PPARγ mRNA during reperfusion may be a compensatory response and increased PPARγ immunoreactivity may be mainly located in the cytoplasm.

Transient focal cerebral ischemia results in an irreversibly damaged ischemic core and salvageable surrounding tissue, penumbra. Cell death in the penumbra is an active process largely dependent on the activation of cell death programs leading to apoptosis [23]. In the present study, low-dose alcohol consumption significantly reduced DNA fragmentation in the peri-infarct cortex. In addition, low-dose alcohol exposure protected the neurons from OGD/reoxygenation-induced apoptosis. Our findings suggest that reduced apoptosis may be also involved in the neuroprotective effect of low-dose alcohol consumption. Two recent studies found that treatment with PPARγ agonists can reduce neuronal apoptosis following transient focal cerebral ischemia [8], [24]. In the present study, GW9662 inhibited the anti-apoptotic effect of low-dose alcohol exposure in cultured neurons. Thus, the anti-apoptotic effect of low-dose alcohol consumption may be related to the upregulation of PPARγ. Interestingly, a previous study found that treatment with PPARγ agonist reduced infarct size in transient but not permanent focal cerebral ischemia [25]. When ischemia is followed promptly by reperfusion, mitochondria produce excessive superoxide [26]. Over production of superoxide blocks mitochondrial respiration and facilitate mitochondrial transition pore formation, which may lead to the release of inner and outer mitochondrial membrane space constituents including cytochrome c and apoptosis-inducing factor (AIF) [27]. Thus, superoxide from mitochondria may play a central role in activating apoptotic pathways following cerebral I/R. PPARγ agonist has been shown to upregulate CuZnSOD in the brain [28]. However, MnSOD is the principal scavenger for superoxide in mitochondria, and therefore of prime importance in maintaining cellular ROS balance and mitochondrial integrity [29]. Thus, important future studies would be to identify the impact of PPARγ activation on MnSOD in the brain.

Cerebral I/R injury is mediated by several overlapping mechanisms, including excitotoxicity, oxidative stress, inflammation and apoptosis. Glutamate transporters/excitatory amino acid transporters (GLTs/EAATs), but especially GLT1/EAAT2, control the extracellular glutamate concentration below excitotoxic levels [30]. We previously found that low-dose alcohol consumption significantly upregulated GLT1/EAAT2 in cerebral cortex and treatment with memantine, a NMDA receptor antagonist, failed to further protect against cerebral I/R injury in low dose alcohol-fed, suggesting that reduced excitotoxicity may be involved in the neuroprotective effect of low-dose alcohol consumption [6]. Two recent studies suggest that GLT1/EAAT2 may be a target gene of PPARγ [31], [32]. Thus, low-dose alcohol consumption may protect against cerebral I/R injury via PPARγ-mediated upregulation of GLT1/EAAT2.

Alcohol consumption has been shown to alter PPARγ expression/activity in organs, tissues and cells. Fortunato et al. found that high-dose alcohol consumption upregulates PPARγ in the pancreas [9]. Chavez et al. reported that high-dose alcohol consumption upregulate PPARγ in the liver [11]. In contrast, Mitra et al. reported that high-dose (100 mM) alcohol exposure downregulated PPARγ in human hepatoma cells [10]. Sun et al. recently found that high-dose alcohol consumption downregulated PPARγ in adipose tissue [12]. In the present study, low-dose alcohol consumption upregulated PPARγ in cerebral cortex. In addition, low-dose (1 and 5 mM) alcohol upregulated, whereas high-dose (10 and 50 mM) alcohol downregulated PPARγ in cultured CATH.a neurons. Thus, dose-dependent and region-related differences may exist in the influence of alcohol consumption on PPARγ expression/activity. In the future, it will be important to determine the mechanisms by which alcohol consumption alters PPARγ expression/activity in the brain.

There are several limitations in the present study. First, while a lot of people under 65 have strokes, ischemic stroke is common among the elderly. Since young animals were used in our studies, it would be necessary to further evaluate the effect of low-alcohol consumption on cerebral I/R injury in aged animals. Second, while isoflurane has been commonly used as an anesthetic agent in mouse models of MCAO, isoflurane has at least short-term neuroprotection in this type ischemic model. Thus, it would be important to determine whether there is an interaction between isoflurane and low-dose alcohol consumption in ischemic stroke.

In summary, the present study further defines the influence of low-dose alcohol consumption on cerebral I/R injury. We suggest that upregulated PPARγ may be involved in the protective effect of low-dose alcohol consumption.

## Materials and Methods

### Animal models of chronic alcohol consumption

All procedures were in accordance with the “Principle of Laboratory Animal Care” (NIH publication no. 86-23, revised 1985) and were approved by the Institutional Animal Care and Use Committee (IACUC) of Louisiana State University Health Sciences Center-Shreveport and University of Nebraska Medical Center. At 2 months of age (body weight 20 to 25 g), male C57BL/6 J mice (n = 48) were divided into six groups, nonalcohol (n = 12), nonalcohol+rosiglitazone (n = 6), nonalcohol+GW9662 (n = 6), 1% alcohol (n = 12), 1% alcohol+rosiglitazone (n = 6), 1% alcohol+GW9662 (n = 6). Mice were fed a liquid diet with or without 1% (v/v) alcohol (Dyets, Bethlehem, PA) for 8 weeks. Nonalcohol diet is 1.0 kcal/ml, of which 35% are derived from fat, 18% are derived from protein, and 47% are derived from carbohydrates. Alcohol diet is 1.0 kcal/ml, of which 35% are derived from fat, 18% are derived from protein, 42% are derived from carbohydrates, and 5% are derived from alcohol. To measure blood alcohol concentration, mice were fasted for 8 hours, blood samples were collected at 0.5, 1, 2 and 4 hours after giving alcohol diet. Plasma alcohol concentration was measured using a Perkin-Elmer gas chromatograph. From the 7^th^ week, rosiglitazone (20 mg/kg/day) and GW9662 (3 mg/kg/day) were mixed into the liquid diet and respectively given to rosiglitazone-treated mice and GW9662-treated mice for two weeks.

### Transient focal cerebral ischemia

Mice were subjected to unilateral MCAO for 90 minutes using the intraluminal filament technique. To avoid any acute effect of alcohol, alcohol-fed mice were given nonalcohol diet 12 hours prior to and after the MCAO. On the day of the experiment, mice were anesthetized with isoflurane (induction at 5% and maintenance at 1.5%) in a gas mixture containing 30% O_2_/70% N_2_ via a facemask. Rectal temperature was maintained at 37°C using a temperature controlled heating pad (TC-1000 Temperature Controller, CWE). A Laser–Doppler flow probe (PeriFlux System 5000, Perimed) was attached to the right side of the dorsal surface of the skull (2 mm caudal and 5 mm lateral to the bregma) to monitor regional cerebral blood flow (rCBF). A 6/0 monofilament nylon suture was prepared by rounding its tip and coating with silicon. The right common and external carotid arteries were exposed and ligated. The middle cerebral artery (MCA) was occluded by inserting the filament from the basal part of the external carotid artery and advancing it cranially into the internal carotid artery to the point where the MCA branched off from the internal carotid artery. Onset of the MCAO was determined by a rapid drop in rCBF. After the right MCA was occluded for 90 minutes, reperfusion was initiated by removing the suture. Animals were allowed to recover for 24 hours.

### Assessment of brain injury

Neurological deficits were evaluated at 24 hours of reperfusion using a 5-point neurological score: 0 = no neurological dysfunction; 1 = flexion of torso and of contralateral forelimb on lifting of the animal by tail; 2 = circling to the contralateral side but normal posture at rest; 3 = leaning to contralateral side at rest; 4 = no spontaneous motor activity. After neurological evaluation, mice were euthanized with Inactin (150 mg/kg body weight) and exsanguination. The brains were quickly removed and placed in ice-cold saline for 5 minutes, and cut into six 1 mm-thick coronal sections. Sections were stained with 2% 2,3,5-triphenyltetrazolium chloride (TTC) for 20 minutes at 37°C. Slice images were digitalized, the infarct lesion was evaluated using Kodak Molecular Imaging Software. Complete lack of staining is defined as infarct lesion. Infarct lesions corrected for cerebral edema were expressed as percentage of the contralateral hemisphere.

### Sample processing

Twelve mice (nonalcohol (n = 6), alcohol (n = 6)) were euthanized with Inactin (150 mg/kg body weight) and exsanguination and the brains were quickly removed. Two 2-mm-thick coronal brain slices, the posterior margin of which is located 2 mm caudal to the optic chiasm, were sectioned. Under microscope, infarct core was identified as opaque area, and the cortex bordering 2 mm the infarct core was considered as the peri-infarct area. Parietal cortex tissues punched at the peri-infarct and contralateral corresponding areas were used for measuring nuclear PPARγ protein/activity and DNA fragmentation.

### DNA fragmentation

Apoptosis was evaluated by quantifying the DNA fragmentation in peri-infarct parietal cortex at 24 hours of reperfusion using a Cell Death detection ELISA kit (Roche Diagnostics, IN) following the manufacturer's protocol.

### CATH.a neuronal cell culture

Catecholaminergic CATH.a neuronal cells (American Type Culture Collection, VA) were cultured in RPMI 1640 medium supplemented with 8% normal horse serum, 4% fetal bovine serum, and 1% penicillin-streptomycin at 37°C in a humidified atmosphere of 5% CO_2_. Before the experiments, CATH.a neurons were differentiated for 6–8 days by addition of *N* ^6^,2′-*O*-dibutyryladenosine 3′,5′-cyclic monophosphate sodium salt (1 mM) to the culture medium. For alcohol exposures, culture media were replaced with media containing 1, 5, 10 and 50 mM alcohol. The alcohol media were changed every 12 hours. After 7-day alcohol exposure, CATH.a neurons were subjected to OGD/reoxygenation experiment or harvested for nuclear PPARγ protein/activity assays.

### OGD/reoxygenation

Neurons exposed 1 mM alcohol alone or in combination with 15 µM GW9662 or 5 µM rosiglitazone for 7 days were subjected to OGD/reoxygenation. To avoid an acute action of alcohol, the last change of alcohol media was performed at 12 hours prior to the OGD and the neurons were cultured without alcohol after the OGD. OGD was induced using a sealed plastic bag aerated with an anaerobic gas mixture (90% N_2_, 5% H_2_, and 5% CO_2_) and kept at 37°C. To initiate OGD, culture media were replaced with deoxygenated, glucose- and alcohol-free medium. After a 2-hour challenge, cultures were removed from the anaerobic bag, and OGD medium in the cultures was replaced with regular medium. Cells were allowed to recover for 24 hours in a regular incubator.

### Assessment of apoptosis

OGD/reoxygenation-induced apoptosis was evaluated using annexin V- fluorescein isothiocyanate (FITC)/propidium iodide (PI) assay kit (AbD Serotec, Raleigh, NC) following the manufacturer's protocol. Briefly, the neurons were harvested and stained with annexin V-FITC and PI for 20 min at room temperature in the dark. The neurons were then washed twice with PBS, and the fluorescence of the neurons was analyzed by flow cytometry for a cell count of 10000. Neurons stained with Annexin-V only were considered as apoptotic cells.

### Nuclear protein extraction

Cortex tissues and cultured neurons were gently homogenized in ice-cold resuspension buffer containing 20·mM HEPES-KOH (pH 7.5), 10· mM KCl, 1.5·mM MgCl_2_, 1·mM EDTA, 1·mM EGTA, protease inhibitor cocktail, phosphatase inhibitor cocktail and 8.59% sucrose. Homogenates were centrifuged for 10·minutes at 750×*g* at 4°C. The pellet will be suspended using a nuclear lysis buffer from nuclear protein extraction kit (Fermentas International, MD) to isolate and purify the nuclear fraction. Protein concentration was determined by the Bradford method (Bio-Rad) with BSA as the standard.

### Western Blot

SDS polyacrylamide gel electrophoresis (SDS-PAGE) was performed on a 10% gel on which 20 µg of total protein per well was loaded. After SDS-PAGE, the proteins were transferred onto polyvinylidene difluoride membrane. Immunoblotting was performed with the use of rabbit anti-PPARγ and mouse anti-proliferating cell nuclear antigen (PCNA) (PC10) (Santa Cruz, CA) as primary and peroxidase conjugated mouse anti-rabbit and goat anti-mouse IgG as the second antibody. The bound antibody was detected by enhanced chemiluminescence (ECL) detection (Pierce Chemical, IL) and the bands were analyzed using UVP BioImaging Systems. For quantification, nuclear PPARγ protein was normalized to the expressed nuclear marker, PCNA.

### PPARγ DNA-binding activity

PPARγ DNA-binding activity was determined by PPARγ transcription factor assay kit (Cayman, MI) following the manufacturer's protocol. In brief, 120 µg nuclear proteins were incubated with a biotin-labelled DNA probe containing a PPAR-specific double-stranded consensus sequence and a single-stranded capture region. The samples were digested with a double-stranded DNA-specific nuclease and subsequently transferred to a 96-well plate coated with single-stranded DNA complementary to the capture region. A chemiluminescent alkine phosphatase substrate was added, and the output signal was measured using a microplate luminometer.

### Statistical analysis

For comparison of the various treatments, results were compared using a two-way repeated measure ANOVA with Turkey's post hoc test. Student *t* tests were used to compare DNA fragmentation and nuclear PPARγ protein/activity before and following chronic exposure to alcohol. Values are means ± SEM. A p value of 0.05 or less was considered to be significant.
